# Is laparoscopic approach safe for radical nephrectomy for tumors larger than 7 cm?

**DOI:** 10.4103/0970-1591.44275

**Published:** 2008

**Authors:** Paresh Jain, Anil Mandhani

**Affiliations:** Department of Urology, Sanjay Gandhi Post Graduate Institute of Medical Sciences, Lucknow, Uttar Pradesh, India

## INTRODUCTION

Laparoscopic radical nephrectomy (LRN) for renal cell carcinoma is said to be the gold standard approach to treat localized renal cell carcinoma but the literature lacks evidence to support this notion.

In medicine, a gold standard is a benchmark that is regarded as definitive. Similarly, a standard of care is a treatment process that a clinician should follow for a certain type of patient, illness, or clinical circumstance. A medical practitioner who does not treat a patient in accordance with the accepted gold standard in his community is often viewed as negligent or even guilty of malpractice. Practice of medicine is evidence-based and evidence is tenuous to generalize the laparoscopic approach to be a standard of care for all renal tumors irrespective of the size.

There have been randomized controlled trials (RCT) comparing early outcome of laparoscopy with open approach and between transperitoneal and retroperitoneal approach but unfortunately no RCT or even well conducted case control trials or cohort studies are available to describe the oncological safety of LRN. Most of the case series are retrospective in nature comparing the open and laparoscopic approach with the inherent bias of choosing smaller tumors in laparoscopic approach.

The immediate benefits of laparoscopy are well established and include less estimated blood loss, decreased pain, shorter perioperative convalescence, and improved cosmesis.

Most of the studies with long-term follow-up on T1 -T2 tumors report a uniform outcome on cancer control for both T1 and T2 but the percentage of T2 tumors treated ranged from 4-11%.[1–4] All these studies are retrospective uncontrolled studies with clear predilection of choosing smaller size tumor for laparoscopy. Surprisingly, in the best of hands there is a risk of tumor violation in laparoscopy for smaller tumor owing to the basic nature of lifting the tumor with rigid instruments, this risk presumably would be more in large tumors.[5] In India the spectrum of renal cell carcinoma is different from the rest of the world where around 60% present with tumor size of more than 7 cm.[6] Therefore there is a need for a stronger evidence to make LRN a standard of care approach.

In a study with the longest follow-up, 10-year disease-free, cancer-specific, and actuarial survival rates were reported as 94%, 97%, and 76% in LRN (mean tumor size 5.1 cm) and 87%, 86%, and 58% for open radical nephrectomy (ORN) (mean tumor size 5.4 cm).[3] In another retrospective study from Cleveland clinic with mean tumor size of 5.4±2.5 cm in laparoscopic and 6.4±2.9 cm in open group, with mean follow up of 60 and 70 months, five-year survival was comparable[4] [Table 1]. One patient in the lap group had positive surgical margin. T2 tumor was present in just 4% of the cases. In another large study from Japan, with median follow-up of 40 months for 195 LRN in T1 renal cell carcinoma with mean tumor size of 3.67±1.3 cm the five-year cancer-specific survival was 91% in comparison to 87% with corresponding 68 patients with tumor size of 4.35 ±1.4 cm in open radical nephrectomy with median follow-up of 67 months.[2

**Table 1:** Recent major studies on laparoscopic radical nephrectomy

Study	Number in lap arm	Mean tumor size (cm)	Follow-up (years)	Five-year survival Lap/open(%)	Type of survival
Portis 2002[1]	64	4.3	4.5	98/92	Projected
Saika 2003[2]	195	3.6	3.3	94/94	Projected
Permpongkosol 2005[3]	121	5.1	6	97/89	Actual
Colombo 2007[4]	45	5.4	5	92	Actual

In one multicenter experience, though the five-year actuarial survival was comparable, mean tumor size in open group was 6.2 cm whereas it was 4.3 cm in the lap group (*P* 0.001).[1] There were only nine tumors greater than 7 cm in the laparoscopic versus 24 in the open group. When mass size was categorized relative to the 4 and 7 cm criteria, the laparoscopic group had more lesions smaller than 4 cm and fewer lesions larger than 7 cm relative to the open group.[1]

Long-term oncologic outcomes for smaller tumors are known based on Level 3 and 4 evidence but the evidence is weak for LRN for large tumors. Patients with LRN for 7-cm or greater masses had a longer operative time and larger increase in postoperative creatinine than patients with less than 7-cm masses.[7] Laparoscopic radical nephrectomy for larger tumors is more difficult than the smaller lesions as there is less working space, more likelihood of nodal disease, distortion of anatomy, displacement of normal organs and difficult hilar dissection.[7] Larger tumors would require retraction with force and thereby there is a greater chance of violation of surgical planes.[7] Margin positivity is reported as 5.7% which is nil in open approach.[8]

In another study comparing LRN for larger and smaller than 7 cm tumors, conversions (1% versus 12%, *P* −0.013) and intraoperative complications (5% versus 19%, *P* − 0.006) were more likely in patients with clinical Stage T2 tumors (9.7±2.5 cm) with margin positivity of 2%. Sixty per cent of conversions in larger tumors were not because of complications but based on the purpose of performing optimal cancer surgery.[9]

Since Clayman *et al.*, described the first LRN 17 years back, there is only one propsective non-randomized study comparing long term outcome between open and LRN in tumors more than 7cm.[10] Hemal *et al.* recently published the only long-term prospective non-randomized comparison of open versus laparoscopy for large renal tumors (T2N0M0).[10] Five-year overall, cancer-specific, and recurrence-free survival were comparable in the two groups. Conversion rate was 5% and there was no local recurrence at 51.4 months of follow-up. In a few patients (number not mentioned) dissection near the renal hilum was difficult because the renal mass prevented direct access to the renal vessels. In such cases after control of the gonadal vein the lower kidney pole was mobilized, followed by posterior dissection, which allowed the lower pole to be lifted up to dissect and control the renal vessels.[10]

Real life practice is not statistics. For an individual it is all or none phenomenon. In cancer surgery to give 10 days of comfort by choosing laparoscopy indiscriminately is in no way better than giving a good 10 years of life. More evidence is needed to prove that the laparoscopic approach is oncologically safe for large tumors; till then it is premature to consider LRN as a standard of care in our practice.
